# Analytical Validation of a Spiral Microfluidic Chip with Hydrofoil-Shaped Pillars for the Enrichment of Circulating Tumor Cells

**DOI:** 10.3390/bios13100938

**Published:** 2023-10-19

**Authors:** Begum Sen-Dogan, Mehmet Alper Demir, Buket Sahin, Ender Yildirim, Gizem Karayalcin, Sebnem Sahin, Ege Mutlu, Taylan Berkin Toral, Ebru Ozgur, Ozge Zorlu, Haluk Kulah

**Affiliations:** 1 Mikro Biyosistemler A.S., 06530 Ankara, Turkey; 2 Department of Mechanical Engineering, Middle East Technical University, 06800 Ankara, Turkey; 3 METU MEMS Center, 06530 Ankara, Turkey; 4 Department of Electrical and Electronics Engineering, Middle East Technical University, 06800 Ankara, Turkey

**Keywords:** microfluidic channel, computational fluid dynamics, circulating tumor cell (CTC) separation, inertial hydrodynamics

## Abstract

The isolation of circulating tumor cells (CTCs) from peripheral blood with high efficiency remains a challenge hindering the utilization of CTC enrichment methods in clinical practice. Here, we propose a microfluidic channel design for the size-based hydrodynamic enrichment of CTCs from blood in an epitope-independent and high-throughput manner. The microfluidic channel comprises a spiral-shaped part followed by a widening part, incorporating successive streamlined pillars, that improves the enrichment efficiency. The design was tested against two benchmark designs, a spiral microfluidic channel and a spiral microfluidic channel followed by a widening channel without the hydrofoils, by processing 5 mL of healthy blood samples spiked with 100 MCF-7 cells. The results proved that the design with hydrofoil-shaped pillars perform significantly better in terms of recovery (recovery rate of 67.9% compared to 23.6% in spiral and 56.7% in spiral with widening section), at a cost of slightly lower white blood cell (WBC) depletion (depletion rate of 94.2% compared to 98.6% in spiral and 94.2% in spiral with widening section), at 1500 µL/min flow rate. For analytical validation, the design was further tested with A549, SKOV-3, and BT-474 cell lines, yielding recovery rates of 62.3 ± 8.4%, 71.0 ± 6.5%, and 82.9 ± 9.9%, respectively. The results are consistent with the size and deformability variation in the respective cell lines, where the increasing size and decreasing deformability affect the recovery rate in a positive manner. The analysis before and after the microfluidic chip process showed that the process does not affect cell viability.

## 1. Introduction

Cancer metastasis, which is the primary cause of mortality in cancer patients, is a multistage process including the detachment of tumor cells from the primary site, intravasation into the bloodstream, arresting at a secondary site, extravasation, and colonization to form metastatic tumors [1]. Detached cancer cells that enter the bloodstream are called circulating tumor cells (CTCs). Due to their key role in the metastatic process, CTCs are considered the most promising liquid biopsy biomarker for cancer diagnosis, prognosis, prediction, stratification, and pharmacodynamics [2,3,4]. CTCs are especially critical for obtaining information on tumor evolution during therapy monitoring, as frequent tissue biopsies are difficult or sometimes impossible to obtain from patients for evaluating the response to cancer therapy. Additionally, CTCs represent a dynamic and heterogeneous cell population arising from multiple metastatic lesions that may change significantly during the disease and course of the therapy. Therefore, each blood sample provides a snapshot of the disease status, which is not possible with standard tissue biopsy. With the current advances in molecular analysis techniques including single-cell analysis at the genomic, transcriptomic, and proteomic levels, the potential of CTC liquid biopsies for the improvement in cancer diagnosis and therapy materializes at a faster pace than before [5,6,7]. Currently, there are around 200 active clinical trials on CTCs with increasing interrogation of CTC molecular characterizations including mutations, epigenetic changes, multigene expression, and protein expression analyses, besides the usual CTC enumeration as the primary measure [8].

Despite its great potential in improving cancer care, the isolation of extremely rare CTCs from peripheral blood cells with high efficiency is still the main challenge that impedes their use in routine clinical practice. Due to their rarity, analysis of CTCs from blood samples without a prior enrichment step is too costly and time-consuming, if not impossible, as the blood volume required to analyze these is relatively high (≥7.5 mL, according to the FDA-approved CellSearch platform) and contains billions of cells which would require too many reagents, slides, and other materials for standard downstream analysis techniques. Over the past decade, a number of technologies have been developed to isolate or enrich CTCs based on their biological and/or physical properties that are distinct from normal hematological cells [9,10,11,12]. Among these, microfluidic approaches for the size-based enrichment of CTCs offer the advantage of epitope-independent, label-free isolation of CTCs from whole blood, providing a more heterogeneous CTC output.

Microfluidic technologies improve the separation efficiency of CTCs due to their innate properties like the small size that is comparable to the size of biological particles, which enables the separation of the CTCs solely based on hydrodynamic forces. Such examples include pinched-flow fractionation [13], deterministic lateral displacement [14,15], asymmetrical curved microchannels [16,17], or spiral microchannels [18], which have been adopted so far to isolate CTCs from blood. Different strategies have been adapted to these methods to further enhance isolation efficiency. For instance, hydrodynamic separation in spiral microchannels have been featured by utilizing a sheath flow [19], by slanting the channel top wall [20], or by reversing the secondary flow direction [21].

In this study, we present a novel spiral microfluidic channel design with a widening outlet section which also includes successive hydrofoil structures, enhancing the size-based separation efficiency of CTCs. The operation of the system does not rely on the existence of a sheath flow, simplifying the overall operation and increasing the throughput. The performance of the system was verified numerically and optimized experimentally. The analytical validation of the system was carried out by spiking experiments using blood samples from healthy donors. Results indicate a high CTC recovery rate independent of the type of cancer cells spiked with no damage to cells in terms of integrity and viability.

## 2. Materials and Methods

### 2.1. Microfluidic Design

Archimedean spirals are often used for the inertial focusing of particles in a stream by utilizing the drag forces induced by Dean vortices across the channel along with the lift forces. The balance between the lift forces ($F_{L}$ in Equation (1)) and the Dean forces ($F_{D}$ in Equation (2)) causes the particles to migrate to a focal position across the channel.

(1) $$F_{L}=\left(4\rho U^{2}a^{2}C_{L}\right)/D_{h}$$

(2) $$F_{D}=3\pi \mu aU_{D}$$

In Equations (1) and (2), a is the particle diameter; U is the flow velocity; μ and ρ are the viscosity and density of the liquid, respectively; and $D_{h}$ is the hydraulic diameter given for rectangular channels by $D_{h}=\left(2wh\right)/\left(w+h\right)$, where w and h are the width and the height of the channel, respectively.

It should be noted that the lift force on a particle is affected by the lift coefficient $C_{L}$, which is a function of the Reynolds number of the flow (Re=ρUDh/μ) and the location of the particle in the channel. $F_{D}$ can be interpreted as the Stokes drag on the particle in the Dean vortex with average Dean velocity, $U_{D}$, which can be estimated using the relation $U_{D}=1.8\times 10^{-4}{\mathrm{De}}^{1.63}$ proposed by Ookawara et al. [22], where De is the Dean number given by

(3) $$\mathrm{De}=\frac{\rho UD_{h}}{\mu }\sqrt{\frac{D_{h}}{2R}}$$

where R is the radius of the curvature of the spiral channel.

**Figure 1:**
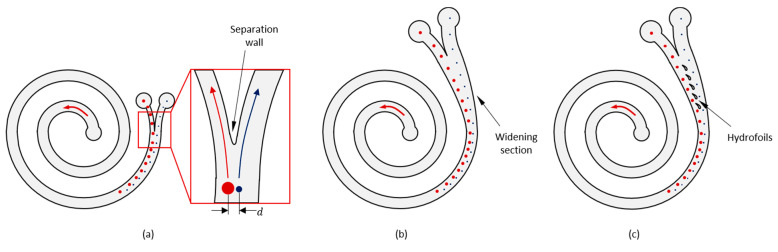
(**a**) Typical Archimedean spiral microfluidic channel with separation wall at the outlet section. (**b**) Spiral microfluidic channel with widening outlet section. (**c**) Spiral microfluidic channel with widening outlet section and hydrofoils.

Particles of different sizes focus along separate streamlines in a spiral channel based on the above theory and then are typically collected at the outlet of the spiral channel for further downstream analyses. A common approach is to locate two or more outlet channels separated from each other considering the loci of the upcoming particles (Figure 1a). However, as the difference between the sizes of the particles becomes smaller, the separation distance between the loci of the particles (d in Figure 1a) also decreases. In such cases, the outlet design becomes as important as the spiral design itself. Mihandoust et al. showed that the performance of a slanted spiral could be improved by properly modifying the outlet geometry [23]. Here, we propose to gently widen the spiral channel at the outlet section (Figure 1b) and pose successive streamlined pillars in the shape of asymmetric hydrofoils upstream of the separation tip, as illustrated in Figure 1c.

To design the spiral channel and the widening outlet section with the hydrofoils, we utilized a hybrid approach combining analytical, numerical, and empirical solutions. Considering the limitations imposed by the fabrication method and the operating conditions explained in Section 2 (the Materials and Methods section), we started by setting the depth of the channel to 80 µm and the flow rate to 1500 µL/min. For the given depth and the flow rate, we searched for a set of feasible channel width and radius of curvature combinations that would allow 10 µm diameter and 14 µm diameter target particles (representing the white blood cells (WBCs) and circulating tumor cells (CTCs), respectively) to focus on definite loci along the spiral. In choosing the feasible set, the confinement ratio, λ, which is defined as the ratio between the particle diameter and the hydraulic diameter ($\lambda =a/D_{h}$), and the Dean number were set as the constraints. For curved channels, the minimum threshold for the confinement ratio for inertial focusing to take place was stated by Martel and Toner to be 0.07 (λ>0.07) [24]. For $a_{1}=10$ µm and $a_{2}=14$ µm diameter particles, we aimed both $\lambda_{1}$ and $\lambda_{2}$ to be greater than 0.07 to ensure the focusing of both sizes of particles. Additionally, the Dean number should be sufficiently large for the Dean vortices across the channel to be effective to focus the particles within a feasible length of the spiral. On the other hand, Nivedita et al. showed that there is a critical Dean number, above which the primary Dean vortices are perturbed and secondary vortices form, which affects the focusing of the particles [25]. This critical Dean number is dependent on the aspect ratio of the channel, and for low-aspect ratio channels (0.2–0.6), it was reported as ranging between ~30 and 40 [25]. Accordingly, we set the feasible Dean number region to 10<De<30. Based on these constraints ($\lambda_{1}>0.07$, $\lambda_{2}>0.07$, 10<De<30), we found a set of channel-width and radius-of-curvature combinations. Within this set, we arbitrarily selected the channel width (w) and the average radius of curvature (R) as 250 µm and 4.5 mm, respectively (R is 4 mm at the inlet and 5 mm at the outlet), which resulted in De=16.7, $\lambda_{1}=0.0825$, and $\lambda_{2}=0.1155$. In the following stages of the design, we further justified this selection by utilizing empirical and numerical models.

**Figure 2:**
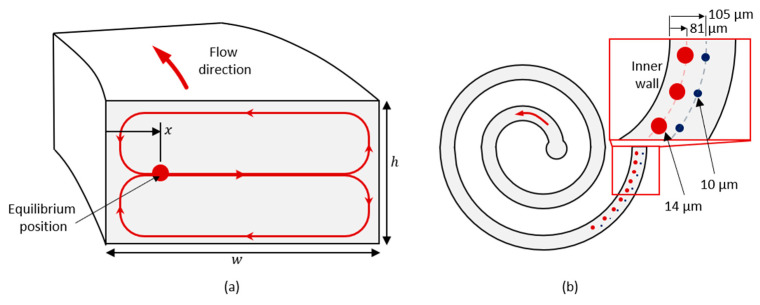
(**a**) Dean vortex across the channel and the equilibrium position of a particle. (**b**) Lateral equilibrium positions of 10 µm and 14 µm diameter particles at the exit of the spiral channel.

To determine the length of the spiral channel, we considered the time passed for the particles to migrate to their respective loci. The particles entering the spiral channel at an arbitrary location at the inlet section drift along the Dean vortices across the channel at average Dean velocity ($U_{D}=1.8\times 10^{-4}{\mathrm{De}}^{1.63}$) until they eventually reach their equilibrium positions. The maximum distance that a particle drifts across the channel before reaching its equilibrium position can be estimated as 2(w+h/2) (Figure 2a). Noting that the particle simultaneously streams along the channel at a flow velocity of U=Q/(wh), the minimum length of the spiral channel that allows the particles to reach their equilibrium positions can be estimated as

(4) $$L_{\mathrm{sp},\min}=\frac{Q(2w+h)}{U_{D}wh}$$

For selected channel dimensions and the flow rate, this length corresponds to a 2-loop Archimedean spiral with radius of curvature of 4.5 mm.

After deciding the geometry of the spiral channel, we estimated the loci of 10 µm and 14 µm diameter particles, respectively. For this purpose, we referred to the experimental data available in the literature [24], where the normalized lateral position of the particles along the width of the channel (*x*/*w*) are presented with respect to non-dimensional parameters, namely the Reynolds number (Re=ρUmaxDh/μ, where $U_{max}$ is 3/2 of the average flow velocity U), confinement ratio (λ), and the curvature ratio ($\delta =D_{h}/2R$). We interpolated the empirical data available in [24] to determine the lateral positions of 10 µm and 14 µm diameter particles at the exit of a 2-loop spiral microchannel with 80 µm height, 250 µm width, and radius of curvature of 4.5 mm. As a result, we calculated that under 1500 µL/min flow of aqueous solution, 14 µm diameter particles representing the CTCs and 10 µm diameter particles representing the WBCs exit the spiral microchannel at lateral positions of 81 µm and 105 µm (Figure 2b), respectively, with respect to the inner wall, making the separation distance d=24 µm, which justifies the design of the spiral microfluidic channel.

To analyze the outlet geometry, we utilized a 2-dimensional numerical model by using COMSOL Multiphysics 5.2a. The outlet section was formed by slightly widening the upstream spiral channel. In our previous work [26], we showed that asymmetric hydrofoil-shaped pillars could be used to improve the separation. Accordingly, we located five successive asymmetric hydrofoils (NACA 9730 profile) in the widening section to enhance the separation distance between different-sized particles by keeping the velocity higher along the outer wall of the channel than along the inner wall of the channel, to keep the larger particles near the inner wall at a slower pace. Downstream of the hydrofoils, a separation wall was posed to direct different-sized particles to their respective outlets (product outlet to collect 14 µm diameter particles representing CTCs and waste outlet to collect 10 µm diameter particles representing WBCs).

**Figure 3:**
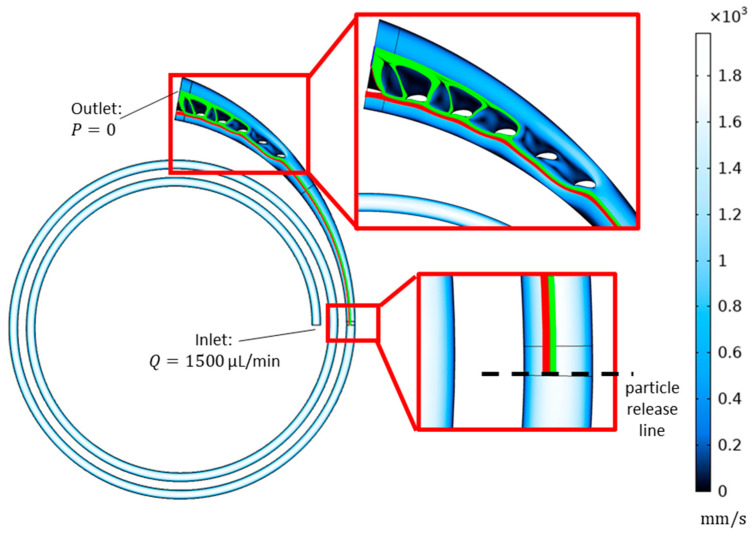
Two-dimensional model for analysis of the widening outlet section with hydrofoils.

Laminar flow and particle tracing modules were used to compute the velocity field and pressure distribution, and the location of the particles at the exit of the outlet section. To include the effect of the upstream channel, not only the outlet geometry but also the spiral microchannel was modeled. On the other hand, utilizing the abovementioned empirical model eliminated the need for a 3-dimensional model that would be required to determine the particle locations. Instead, we computed the particle trajectories by releasing 10 µm and 14 µm diameter particles at lateral positions of 81 µm and 105 µm, respectively, at the exit of the spiral section. Figure 3 illustrates the model and the boundary conditions.

### 2.2. Microfluidic Chip Fabrication

The microfluidic chips were fabricated with a MEMS-based silicon glass stack process where the microfluidic channels were formed on the silicon side. In the beginning, the microfluidic channel pattern was formed on the active side of the silicon wafer through DRIE etching with a target depth of 80 µm, through a photoresist mask. After the channel formation, the silicon wafers were cleaned and coated with thermal oxide, and further with PECVD oxide at the active side as a protection layer. The microfluidic inlet and outlet ports were then formed with a second DRIE process, which was applied from the backside of the wafer along its thickness using the thermally deposited SiO_2_ as a masking layer as well as the photoresist used for patterning the SiO_2_. This was followed by cleaning and oxide stripping processes. Then, 0.5 µm of sacrificial thermal oxide was successively deposited and stripped to remove possible residues and scallops formed on the channel and port walls during the DRIE processes. Afterward, the silicon wafer was coated with 0.3 µm thick thermal oxide and was anodically bonded with a blank glass wafer, closing the microfluidic channel and forming a transparent window. The process ended with the dicing of the wafers into chips. Channel sections were analyzed with SEM at 1.0 kV, x30 (METU MEMS Center).

### 2.3. Cell Culture, Blood Collection, and Sample Preparation

Cultured human breast cancer MCF-7 and BT-474 cell lines, the human non-small-cell lung cancer A549 cell line, and the human ovary adenocarcinoma SKOV-3 cell line were obtained from American Type Culture Collection (ATCC, Manassas, VA, USA). All cell lines were cultured at 37 °C in 5% CO_2_. The MCF-7 and SKOV-3 cell lines were cultured in a growth medium containing Dulbecco’s Modified Eagle’s Medium—High Glucose (DMEM-HG) (Biological Industries, Kibbutz Beit HaEmek, Israel), 10% fetal bovine serum (FBS) (Biological Industries, Kibbutz Beit HaEmek, Israel), 1% minimum essential medium (MEM), non-essential amino acids (Biological Industries, Kibbutz Beit HaEmek, Israel) and 1% penicillin–streptomycin ( Biological Industries, Kibbutz Beit HaEmek, Israel). The A549 cell line was cultured with DMEM-HG, 10% FBS, and 1% penicillin–streptomycin. The BT-474 cell line was cultured with RPMI-1640 medium (Biological Industries, Kibbutz Beit HaEmek, Israel), 10% FBS, 1% penicillin–streptomycin, 1% MEM non-essential amino acids, and 0.01% insulin (Lonza, Verviers, Belgium).

Cells were passaged until 70–80% confluency, and then they were detached from the culture flask with 0.25% trypsin-EDTA (Biological Industries, Kibbutz Beit HaEmek, Israel) and resuspended in phosphate-buffered saline (PBS) solution. For analytical performance evaluation, spiking experiments were carried out with cancer cells fluorescently labeled with Cell Tracker Red CMTPX Dye (Invitrogen, Waltham, MA, USA). The cell concentration was measured with a TC20 automated cell counter (BioRAD, Hercules, CA, USA). Trypan blue was used to measure cell viability. To achieve the desired cell number in spiking experiments for analytical performance evaluation studies, cells were diluted with the serial dilution and spiked (20–400 cells/0.5 mL PBS) into whole blood (5 mL) collected from healthy donors.

Ethical approval for blood collection was taken from the Ethical Committee of Zekai Tahir Burak Women’s Health Research and Education Hospital, Ankara, Turkey, and the studies performed were in accordance with the ethical regulations under ethical-committee-approved protocols (Protocol No: MBS-CTC-HEU-AV-02, Date: 27 January 2019). An informed consent form, which was approved by the ethical committee, was obtained from each donor before participating in the study. Blood samples were collected in K_2_EDTA blood collection tubes and processed on the same day.

The spiked whole blood was processed with the density-gradient centrifugation method using Ficoll Paque Plus (Cytiva, Uppsala, Sweden) to eliminate red blood cells (RBCs). The buffy coat containing peripheral blood mononuclear cells (PBMCs) and CTCs was collected and washed with PBS containing 1% FBS (F-PBS). The supernatant was discarded, and the cell pellet was resuspended in F-PBS (10 mL). Before the microfluidic CTC enrichment process, the cell suspension was filtered through a 30 µm cell strainer (Miltenyi Biotec, Bergisch Gladbach, Germany) to remove impurities and prevent clogging.

### 2.4. Experimental Setup

**Figure 4:**
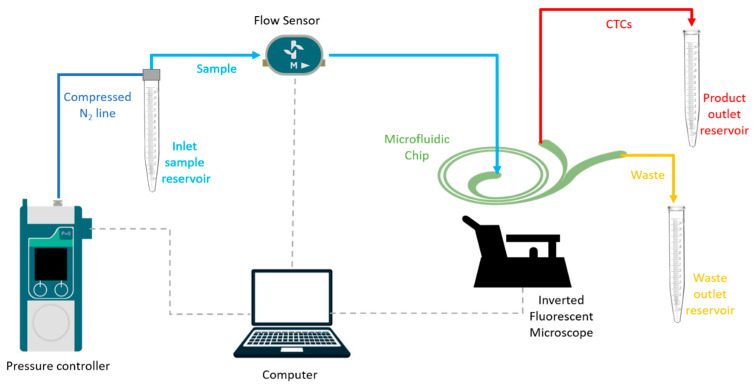
Experimental setup for microfluidic sample processing. The fluid flow is regulated with a flow controller supplied by compressed N_2_ line. Flow rate is adjusted with the feedback control based on the readings of a thermal flow sensor. Channel is observed using an inverted microscope.

The experimental setup for microfluidic sample processing is represented in Figure 4. The fluid flow at a specified flow rate was supplied through a pressure-driven microfluidic setup supplied by a compressed N_2_ line and consisting of a pressure controller (Fluigent Flow EZ 7000, Paris, France) to regulate the pressure to push the liquids at a specified flow rate, a thermal flow sensor (Fluigent Flow Unit XL, Paris, France) for real-time measurement of the flow rate, a custom-design chip holder that enables the connections of the chip to external microfluidic components and to inlet/outlet fluid reservoirs, and an inverted microscope (DMi8, Leica Microsystems, Wetzlar, Germany).

### 2.5. Chip Conditioning and Sample Processing

To prevent biofouling, the inner surfaces of the microfluidic chip and external fluidic components were coated with a random-graft co-polymer with a poly(L-lysine) backbone and poly(ethylene glycol) side chains (PLL-g-PEG Polymer—SuSoS, Zürich, Switzerland). Briefly, the channel was first conditioned with ethanol and washed with deionized (DI) water. Afterward, the channel was filled with PLL-g-PEG (1% in DI water) and incubated for 30 min. Then, the channel was washed with DI water and phosphate-buffered saline (PBS) solution before processing the cell suspension.

The cell suspension was fed to the chip with a constant volumetric flow rate, measured continuously with the flow sensor. A pressure controller regulated the applied pressure to the system with feedback from the flow sensor to maintain a steady flow rate. After the whole sample was processed on the system, the remaining sample in the system’s dead volume (0.68 mL) was flushed with 1 mL of PBS with the same flow rate. The processed sample was collected into two outlets: the product outlet containing the enriched CTC sample and the waste outlet containing blood cells.

### 2.6. Chip Characterization Using Fluorescent Microbeads and Cells

**Figure 5:**
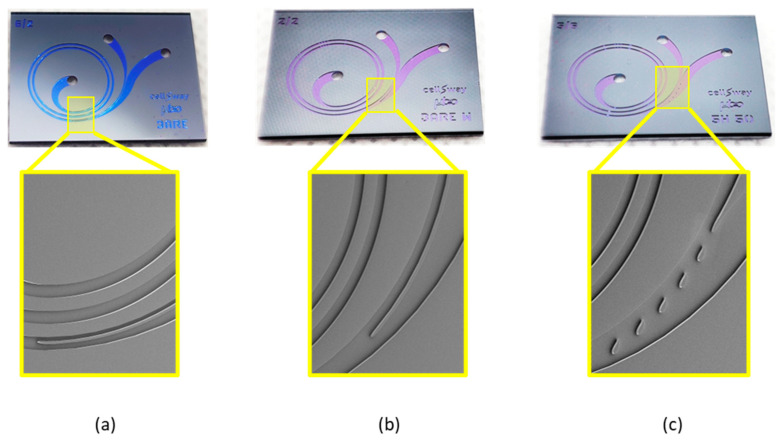
Microfabricated silicon–glass microfluidic chips. (**a**): BARE design, (**b**): BARE-W design, (**c**): 5H-50 design. Insets show the SEM images of separation regions for each chip design.

The microfluidic chip design (namely 5H-50) and the two benchmark designs without the widening channel and without any hydrofoil structure, namely BARE and BARE-W, respectively, were characterized to identify their optimum operating conditions and preliminary performance characteristics. BARE (Figure 5a) was designed as a standard Archimedean spiral with a constant channel width. BARE-W (Figure 5b) has a similar channel geometry to that of 5H-50 but does not contain any hydrofoil structures in the widening channel, while 5H-50 (Figure 5c) contains five consecutive hydrofoils in the widening channel section specifically positioned to increase the performance characteristics, namely the CTC recovery rate and WBC depletion rate.

The CTC recovery rate was calculated by quantifying the number of CTCs at the product and waste outlets through triplicate measurements with the automated cell counter. The outlet suspension volumes and the average CTC concentrations in the waste and product outlet cell suspensions were used to calculate the recovery rate according to the following equation:

(5) $$\mathrm{CTC}\;\mathrm{Recovery}\;\mathrm{rate}\;\left(\%\right)=\frac{\#\;\mathrm{of}\;\mathrm{CTCs}\;\mathrm{at}\;\mathrm{product}\;\mathrm{outlet}}{\#\;\mathrm{of}\;\mathrm{total}\;\mathrm{CTCs}\;\mathrm{at}\;\mathrm{product}\;\mathrm{and}\;\mathrm{waste}\;\mathrm{outlets}\;}\times 100$$

To estimate the WBC depletion rate, total cell concentrations were measured at the inlet and product outlet using the automated cell counter in triplicates. The average WBC concentrations in the inlet and product outlet cell suspensions were used to calculate WBC depletion rate according to the following equation:

(6) $$\mathrm{WBC}\;\mathrm{Depletion}\;\mathrm{rate}\;\left(\%\right)=\left(1-\frac{\#\;\mathrm{of}\;\mathrm{WBCs}\;\mathrm{at}\;\mathrm{product}\;\mathrm{outlet}}{\#\;\mathrm{of}\;\mathrm{WBCs}\;\mathrm{at}\;\mathrm{inlet}\;\mathrm{sample}}\right)\times 100$$

The chip characterization experiments were carried out initially using fluorescent polystyrene microbeads with 10.0 ± 0.6 µm and 18.7 ± 0.7 µm diameters (Polysciences Europe GmbH, Heidelberg, Germany) to represent WBCs and CTCs, respectively, and also using separate suspensions of MCF-7 breast cancer cell lines and WBCs. To identify the optimum flow rate, the bead or cell suspensions (1 × 10^5^ particles/mL) were separately processed on the channel at flow rates varying between 500 µL/min and 2100 µL/min at an increment of 100 µL/min. At least 3 mL of particle suspension was processed at each selected flow rate to ensure that the set flow rate was stabilized. Video recordings were taken under an inverted fluorescent microscope equipped with a high-resolution camera (Hamamatsu ORCA Flash 4.0, Hamamatsu, Japan). The screenshots of the recordings were captured with VLC Video Player software (3.0.12) with a 30 fps capture rate and recording ratio of 2. The fluorescent intensity generated by microbeads across the channel width was analyzed by using the ImageJ software (1.50i) to find the focusing point of the microbeads across the channel. At least three independent experiments per bead and cell type were carried out with each chip design and the results were compared. These studies resulted in the preliminary identification of optimum volumetric flow rates of the chip designs and the initial performance characterization in terms of CTC recovery rates and WBC depletion rates.

### 2.7. Analytical Studies for Design Validation and Performance Characterization

Design validation studies were carried out using MCF-7 breast cancer cells (100 cells) spiked into healthy blood samples. We compared the performances of the proposed 5H-50 design with that of the BARE-W design at the optimum volumetric flow rate determined through initial characterization experiments. At least five independent experiments were carried out with each chip design, and the results were compared in terms of CTC recovery rate, WBC depletion rate, and cell viability at the outlet.

The CTC recovery rate was calculated by quantifying the number of Cell Tracker Red (CTR)-labeled MCF-7 cells. After processing the sample on the microfluidic chip, the cells collected at the product and waste outlets were centrifuged for 5 min and resuspended in 1 mL of PBS, and then seeded in a 96-well plate (100 µL per well) for optical examination. Then, images of the wells were acquired using the inverted fluorescence microscope equipped with a programmable motorized stage. All images were analyzed and MCF-7 cells in each well were automatically enumerated using a custom-designed software (Aurvis, Ankara, Turkey). The recovery rate was calculated according to Equation (5) after determining the total number of CTR-labeled CTCs in each suspension.

Further performance characterization studies were carried out in order to determine the recovery rate of the 5H-50 chip at different MCF-7-cell-spiking rates to show the linearity of the CTC recovery. MCF-7 cells were spiked to the whole blood with serial dilution at a spiking rate covering the range of 10^1^–10^2^ cells, and the blood sample was processed on the microfluidic chip after PMBC isolation. At each spiking rate, at least five experiments were conducted to show reproducibility and linearity between the spiked CTC number and the collected CTC number at the product outlet of the chip.

The applicability of the technology for different cancer types was demonstrated by testing the performance of the 5H-50 design with cancer cell lines derived from different cancer types, including non-small-cell lung cancer (A549, epithelial adenocarcinoma), ovarian cancer (SKOV-3, serous cystadenocarcinoma), and breast cancer (BT-474, ductal adenocarcinoma). The A549, SKOV-3, and BT-474 cell lines were spiked separately into whole blood (5 mL) at a spiking rate of 100 cells per 5 mL of blood. After PMBC isolation, the cell suspension was processed on the chip. At least five experiments were conducted with each cell line and their average recovery and depletion rates were compared with each other.

To investigate the effect of the microfluidic spiral chip process on CTC viability, cultured MCF-7 cancer cells (5 mL, 1 × 10^6^ cells/mL) were processed on the chip at a flow rate of 1500 µL/min. Cell viability at the inlet and outlet cell suspensions was estimated via a Trypan blue (Sigma-Aldrich, Münich, Germany) exclusion assay and analyzed via an automated cell counter. Three independent experiments were carried out and all measurements were conducted in triplicate. The percent viability is defined as the number of live cells over the number of total cells in the inlet and product outlet suspensions, as in the equation given below.

(7) $$\mathrm{Viability}(\%)=\frac{\#\mathrm{of}\;\mathrm{viable}\;\mathrm{cells}}{\#\mathrm{of}\;\mathrm{total}\;\mathrm{cells}}\times 100$$

## 3. Results

This section may be divided into subheadings. It should provide a concise and precise description of the experimental results and their interpretation, as well as the experimental conclusions that can be drawn.

### 3.1. Chip Characterization Using Fluorescent Microbeads and Cells

**Figure 6:**
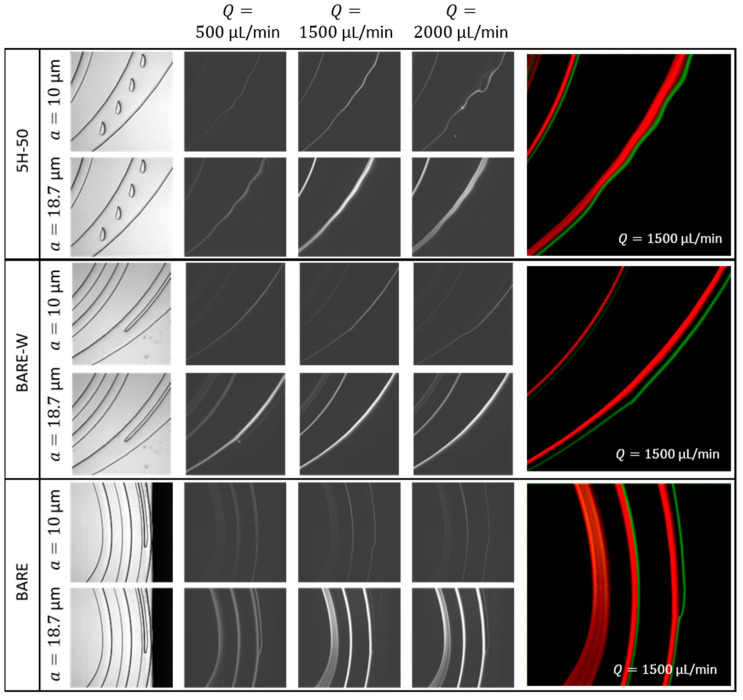
Bright-field and fluorescent images showing the focusing lines for 10 µm beads and 18.7 µm beads along the different channel designs at different flow rates. Fluorescent images were generated by overlaying the pseudocolored bright-field images for 18.7 µm beads (red) and 10 µm beads (green) at a 1500 µL/min flow rate.

To corroborate the design principle and determine the flow conditions, different flow rates were systematically compared with a set of experiments. Firstly, fluorescent polystyrene beads mimicking WBCs and CTCs with 10 ± 0.6 µm and 18.7 ± 0.7 µm diameters, respectively, were tested separately through different microfluidic channel designs to observe the particle streams’ behaviors between 500 and 2100 µL/min. Composite images of particle streams in each flow rate were captured and analyzed using ImageJ software. Figure 6 shows experimental data illustrating the distribution of 10 µm and 18.7 µm beads across 5H-50, BARE-W, and BARE microchannels for the chosen flow rates (Figure S1 for details). For both 10 µm and 18.7 µm particles, the variation in migration pattern with the flow rate were similar, regardless of the channel design. The results showed that 18.7 µm particles were not focused at low flow rates (<1200 µL/min). As the flow rate increased, especially around the design flow rate (1500 µL/min), larger particles tended towards the inner wall, as expected, while at flow rates higher than 1800 µL/min, the focusing was disturbed. On the contrary, 10 µm particles were directed toward the inner wall at low flow rates without a complete focusing. As the flow rate increased, they were directed to the waste outlet near the outer wall, and the focusing was still observed even at flow rates higher than 1800 µL/min. These studies have proven that the designed channels work as expected and the different-sized particles could be collected at the desired outlets by focusing them at different points across the width of the spiral channel. The results also showed that the flow rate range can be adjusted between 1200 µL/min and 1800 µL/min for 5H-50, BARE-W, and BARE channel designs to achieve the separation of particles with specific sizes. However, further increases in flow rate resulted in the dispersion of focus across the channel.

**Figure 7:**
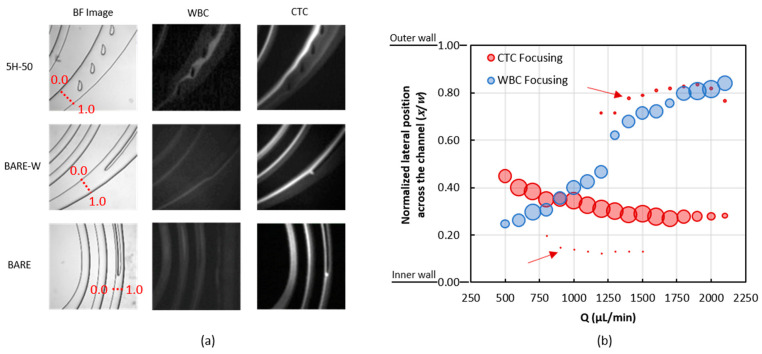
(**a**): Different focusing lines of fluorescently stained MCF-7 cells and WBCs inside 5H-50, BARE-W, and BARE chip designs, together with their corresponding bright-field images at 1500 µL/min flow rate. (**b**): Normalized lateral position (*x*/*w*) of MCF-7 cells and WBCs across the channel of 5H-50. The diameter of the data markers represents the normalized intensity. It is noted that MCF-7 cells were focused at secondary and tertiary positions (indicated by arrows) in addition to the primary equilibrium position. The data for fluorescent intensity distribution along the channel width were extracted on the red dotted lines drawn in the separation region as presented in (**a**).

After characterizing the channels for approximate operating flow rates and achieving proof of focusing, the channels were investigated for the focusing behavior of the MCF-7 cells and WBCs for different flow rates. Figure 7a shows the focusing lines for fluorescent stained MCF-7 cells and WBCs for three different channel designs at 1500 µL/min. Focusing for WBCs near the outer wall and focusing for MCF-7 cells near the inner wall were both observed for all the designs at this flow rate. Red-dotted traces marked on the bright-field images illustrate the focusing point identification lines across the channel width. Figure 7b was generated using the fluorescence intensity profiles of the particle distributions across the channel width for the 5H-50 channel at different flow rates. Focusing points on the plots were normalized to channel cross-sections where the fluorescence readings were taken so that 0.0 marked the inner wall and 1.0 marked the outer wall positions. The focusing point was defined as the distance from the highest fluorescence intensity to the inner wall. Fluorescence for WBCs was observed along a single line and the width of the line was thinner as the focusing occurred around 1500 µL/min. However, two or three focusing lines were observed for MCF-7 cells (Figure 7b), depending on the flow rate. One of these lines—the primary focusing line—had a more intense fluorescence. The other lines—the secondary and the tertiary focusing lines—exhibited a less intense fluorescence (note the line seen near the outer wall of 5H-50 on Figure 7a). These fainter lines may stem from the formation of the secondary Dean vortices at higher flow rates, i.e., at higher Re, as explained by Nivedita et al. in 2017 [25]. These secondary Dean vortices may cause the particles to migrate further and become trapped on an additional focusing line shifted from the inner wall to the outer wall of the channel.

**Figure 8:**
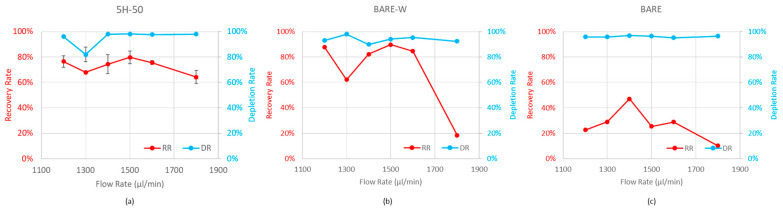
Variation in MCF-7 recovery and WBC depletion rates against flow rate when 1 × 10^5^ cells/mL was used for (**a**) 5H-50, (**b**) BARE-W, and (**c**) BARE (**b**) chips. Optimal results were obtained at the design flow rate of 1500 µL/min in both 5H-50 and BARE-W.

Further experiments were carried out with separate suspensions of WBCs and MCF-7 cells to determine the optimum flow rate in the 1200–1800 µL/min range. Figure 8 shows the depletion and recovery rate performances for WBCs and MCF-7 cells, respectively, at the selected flow rate range for the 5H-50, BARE, and BARE-W channels. According to the results for the 5H-50 design, above 1300 µL/min, the effect of the flow rate on the depletion rate was insignificant. However, the recovery rate of the MCF-7 cells peaked at 1500 µL/min (Figure 8a). For this reason, the optimal flow rate was decided to be 1500 µL/min, which was also the design flow rate used in numerical calculations. At this flow rate, 80% of MCF-7 cells were recovered and 98% of the WBCs were depleted at the CTC outlet. In the BARE-W design, a similar trend was observed in terms of recovery rate. The highest WBC depletion rate was observed at 1300 µL/min, while the highest recovery rate was observed at 1500 µL/min (Figure 8b). Therefore, 1500 µL/min was chosen as the optimum flow rate for the BARE-W design. When Figure 8c was analyzed, it was seen that the benchmark Archimedean spiral had a high WBC depletion rate at all the tested flow rates; however, the maximum MCF-7 recovery at the optimum flow rate of 1400 µL/min could only be around 50%, which is significantly lower than that of the BARE-W and 5H-50 designs.

To investigate the effect of the microfluidic chip process on cell viability, we carried out a viability analysis through a Trypan blue exclusion assay, utilizing MCF-7 cell suspension. The cell viability levels measured at the inlet and outlet cell suspensions were compared together with the total cell count in each suspension to see if there was any cell loss during the process. Cell viability data (obtained using Equation (7)) revealed that the inlet and product outlet cell suspensions have very close total cell viability values (82 ± 6% and 88 ± 5%, respectively), while the waste cell suspension has a slightly lower total cell viability (55 ± 15%). This can be interpreted as meaning that the collection of dead cells was mostly at the waste outlet, which is an expected result as dead cells are typically smaller in size. Total cell counts at inlet cell suspension (6.5 × 10^6^) and outlet cell suspensions (6.2 × 10^6^) revealed that the chip process does not cause significant cell loss (<5%).

**Figure 9:**
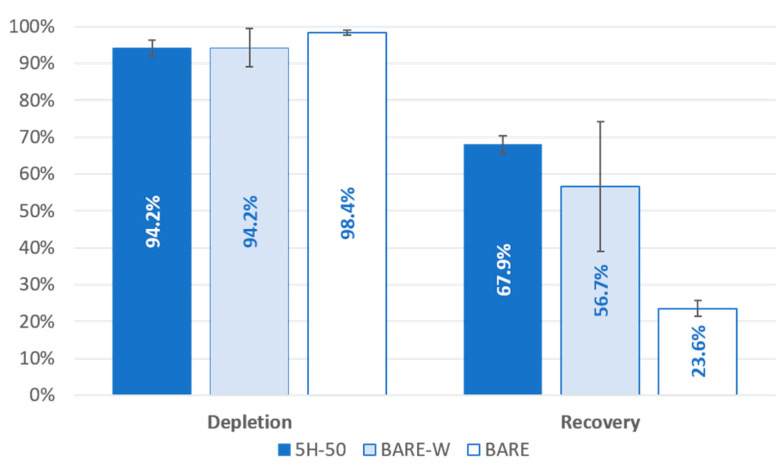
WBC depletion and MCF-7 recovery rates obtained from 5H-50, BARE-W, and BARE chip designs in spiking experiments. Data were collected by spiking 100 MCF-7 cells in 5 mL of whole blood at 1500 µL/min. Data show the average of at least three experiments with standard deviation.

In order to further validate the design, we compared the performances of the 5H-50 chip and the benchmark BARE-W and BARE chips with experiments carried out using MCF-7 breast cancer cell lines spiked into healthy blood samples (100 cells/5 mL blood) at 1500 µL/min. The average WBC depletion and MCF-7 recovery rates are presented in Figure 9. The BARE-W and 5H-50 designs had similar WBC depletion rates of 94.2 ± 2.4% and 94.2 ± 2.2%, respectively. The BARE chip had the highest depletion rate (98.4 ± 0.6%) but the lowest recovery rate (23.6 ± 2.1%). The average CTC recovery rate for the 5H-50 design (67.9 ± 5.2%) was higher than that obtained with the BARE-W design (56.7 ± 17.6%). Additionally, the reproducibility of the results was much better with the 5H-50 chip, as evident from the standard deviations of both data sets. These data show the experimental validation of the numerical design, confirming that the inclusion of hydrofoil structures into a widening spiral microfluidic channel section improves the enrichment of the circulating tumor cells. During the design of the microfluidic channel, the aim of adding hydrofoil structures was to keep the velocity higher along the outer wall of the channel than along the inner wall of the channel to keep the larger particles, in this study, MCF-7 cells, near the inner wall with a slower pace. We confirmed this behavior by reaching a higher recovery rate for the 5H-50 design. It should be noted that the recovery rate and depletion rate values obtained during the spiking experiments for all of the designs were lower than the ones obtained using separate 1 × 10^5^ cells/mL suspensions. This might be attributed to particle–particle interaction being much more effective for the sample used in spiking experiments due to the existence of a high concentration of WBCs inside the solution (>2 × 10^6^). It is possible that the focused flow generated by the much higher number of WBCs may have prevented the CTCs from reaching their equilibrium points.

In light of these results demonstrating the design validation, further analytical studies were carried out only on the 5H-50 design.

### 3.2. Analytical Studies for Design Validation and Performance Characterization

**Figure 10:**
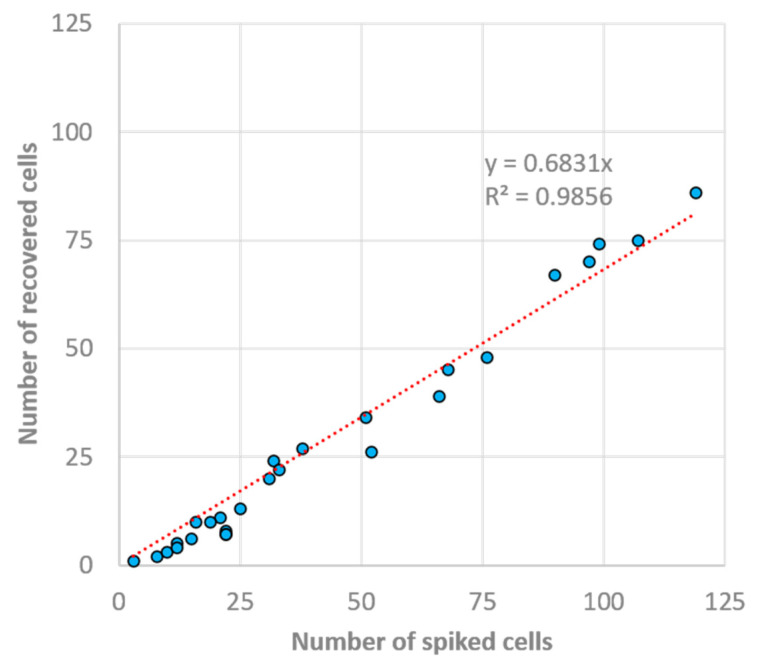
The relationship between spiked and collected MCF-7 cells. Data were generated through 27 independent experiments carried out at varying MCF-7-spiking rates. The linear regression was calculated to be R^2^ = 0.9856.

Analytical validation of the 5H-50 chip design was performed initially with MCF-7 cell lines spiked into healthy blood samples at spiking rates varying between 20 and 400 per 5 mL of whole blood. Figure 10 shows the linear regression between the total number of cells spiked and the cells collected at the product outlet over a total of 27 experiments. The R^2^ value of the regression model was calculated to be 0.9856, showing the high linearity of the MCF-7 cells’ recovery over the experimented spiking rate range. The slope of the line was calculated to be 0.6831, which can be interpreted as an average 68.3% recovery rate of MCF-7s. The average depletion rate obtained from these experiments was 94.8 ± 2.1%. Both the recovery and depletion rates were in good agreement with the ones obtained during the 100 MCF-7 cell spiking experiments. Although the literature reports the purity (recovered CTC amount per contaminating WBC amount) as a metric for the cell separation systems, here, we prefer to report depletion rate since (i) it is more informative as a characterization metric, especially for comparison purposes, and (ii) the purity in clinical samples varies drastically as the numbers of CTCs and WBCs significantly differ from patient to patient.

**Figure 11:**
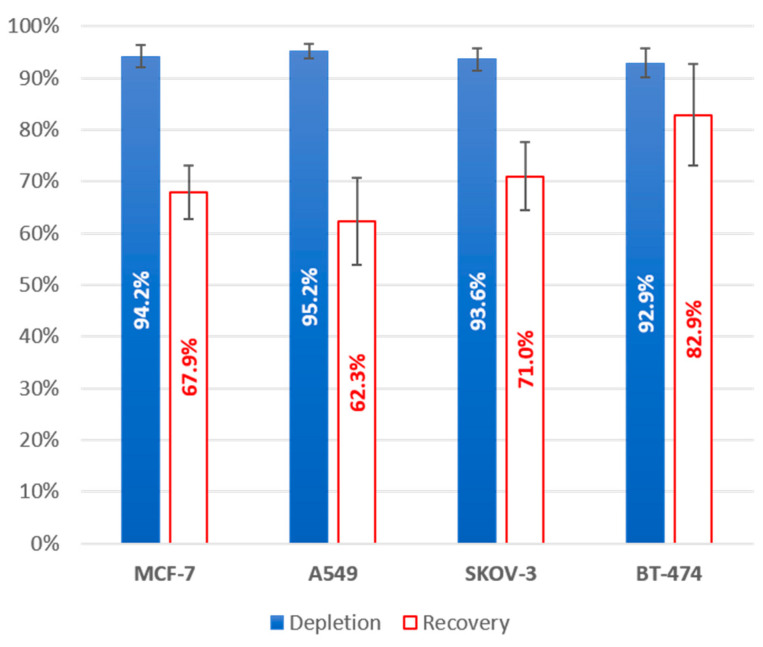
Recovery rates obtained with MCF-7 (n = 8), A549 (n = 5), SKOV-3 (n = 7), and BT-474 (n = 5) cancer cell lines spiked into healthy blood sample (5 mL).

The performance of the 5H-50 chip design was also evaluated with cancer cell lines derived from different cancer types, including lung (A549) and ovarian cancer (SKOV-3), as well as with another breast cancer cell line (BT-474). At the spiking rate of 100 cells/5 mL of whole blood, recoveries above 60% were obtained for all cell lines, reaching a maximum of 82.9 ± 9.9% with BT-474 (Figure 11). The average depletion rate was calculated to be 94.0 ± 1.0%. Difference in the recovery rates mainly depends on the different average cell size of each cell line. Among the studied cell lines, the BT-474 cells (20.9 ± 4.2 µm) were the largest, followed by the SKOV-3 cells (19.9 ± 3.6 µm) and the A549 cells (19.1 ± 4.1 µm). The MCF-7 cells (16.5 ± 2.3 µm) were the smallest. Since the designed microchannel separates cells based on their size differences, the BT-474 cells had the highest recovery (82.9 ± 9.9%), as expected. Although the MCF-7 cells were the smallest, they had a higher recovery rate than the A549 cells. This can be explained by the differences in their deformability, i.e., the alteration in their morphology under stress. It is documented in the literature that A549 cells, which had the lowest recovery (62.3 ± 8.4%), are more deformable than the other three cell lines and BT-474 cells are the least deformable among them [27,28,29]. Thus, we may conclude that the other than particle size differences, the extent of their deformability is also important because shape of the particles affects their migration pattern [30]. A similar effect was also observed when the recovery rate for BT-474 was compared with that of SKOV-3 cells, which are more deformable than BT-474. Although the average cell sizes of these two cell lines are very close, the recovery rate observed for BT-474 cells (82.9 ± 9.9%) was significantly higher than that obtained for SKOV-3 cells (71.0 ± 6.5%). Figure S2 shows the immunofluorescent staining of enriched BT-474 cell suspension, which exemplifies the intactness of the cells processed inside the spiral channel.

## 4. Conclusions

CTCs are considered some of the most promising liquid biopsy biomarkers for obtaining real-time information on tumor evolution during therapy monitoring and cancer management. However, their current use in clinical practice is mainly restricted, used for prognostic stratification and monitoring as opposed to their vast potential. The isolation of extremely rare CTCs from peripheral blood cells with high efficiency is still the main challenge that impedes their extensive use in routine clinical practice as this challenge limits the development of sensitive identification and characterization assays on CTCs.

This paper addresses this challenge with a microfluidic channel design to enhance the size-based enrichment efficiency of viable CTCs from blood. For this purpose, we first developed a design methodology for the channel design by combining analytical, numerical, and empirical solutions. The front end of the channel was designed as a two-turn Archimedean spiral, and a gently widening outlet posing successive streamlined pillars in the shape of asymmetric hydrofoils was designed using 2-D numerical modelling. The single-inlet and two-outlet microfluidic channel operates at high throughput (1500 µL/min) and enables a simpler operation without the need to use sheath flow at the inlet. The performance of the design (namely 5H-50) was analytically demonstrated and benchmarked with two individually optimized designs comprising (i) only an Archimedean spiral (BARE) and (ii) an Archimedean spiral followed by a widening outlet (BARE-W). The optimized flow rate was determined for each design through fluorescent intensity data generated using microbeads and depletion and recovery rate values obtained with WBCs and MCF-7 cell lines. The designs were analytically validated with 100 MCF-7 cells spiked in 5 mL healthy whole-blood samples. It was demonstrated that the 5H-50 design outperformed the benchmark designs with a 67.9 ± 5.2% recovery rate, with a slightly lower depletion rate of 94.2 ± 2.2%. The R^2^ value of the linear fit was measured to be 0.9856 within the MCF-7 spiking range of 10^1^–10^2^ cells per 5 mL. The viability of the cancer cell lines collected at the product outlet was measured to be 88 ± 5%, which is higher than the viability at the inlet. The design was further validated with the spiking of the A549, SKOV-3, and BT-474 cell lines yielding recovery rates above 60%, the highest being recorded as 82.9 ± 9.9% with BT-474 cells. The results indicate a high CTC recovery rate, independent of the type of spiked cancer cells, with no damage to cells in terms of integrity and viability. The method allows the rapid and precise phenotypical characterization of CTCs at DNA, protein, and gene-expression levels through the application of NGS, immunofluorescence, and FISH/RNA-ISH techniques, at a single-cell level. However, for the bulk molecular characterization of isolated cells, the number of WBCs at the product output should be reduced.

Future work will include conducting validation studies with patient blood samples mainly on breast and non-small-cell lung cancer. The studies can also be expanded to cover different cancer types, as the enrichment technique is suitable for use for almost all cancer types except hematological cancers. Moreover, the CTC enrichment workflow will be automated to reduce the hands-on time and to better adapt the developed workflow for use in clinical laboratories. The validation studies will explore both the identification and characterization of enriched CTCs from patient blood samples; hence, they require the integration of immunofluorescent microscopy techniques into the workflow. In our opinion, with the demonstration of the capability of identifying relevant biomarkers on CTCs in a standardized way, this workflow will be a step forward in revealing the clinical utility of CTCs through the generated clinically actionable data to be used for patient follow-up, therapy guidance, and precision medicine.

## Data Availability

Available upon request.

## Supplementary Materials

The following supporting information can be downloaded at: https://www.mdpi.com/article/10.3390/bios13100938/s1, Figure S1: Bright-field and fluorescent images showing the focusing lines for 10 µm beads and 18.7 µm beads along the different channel designs at different flow rates; Figure S2: IF staining of BT-474 cells and white blood cells. BT-474 cells are ER/PR+, HER2+, CK+, DAPI+, and CD45-. Channels; Green: ER/PR, BF: Bright Field, Orange: HER2, Cy5: CD45, Cy7: pan Cytokeratin and DAPI.
