# Supplementary material for: Analytical Validation of a Spiral Microfluidic Chip with Hydrofoil-Shaped Pillars for the Enrichment of Circulating Tumor Cells
Source: Biosensors (Basel). 2023 Oct 19;13(10):938. doi: 10.3390/bios13100938 (PMC10605072; doi:10.3390/bios13100938)
Supplement: Supplementary file 1 [file biosensors-13-00938-s001.zip › biosensors-2609407-supplementary.pdf]

Supplementary Material

Analytical Validation of a Spiral Microfluidic Chip with Hydrofoil-Shaped Pillars for the Enrichment of Circulating Tumor Cells

Begum Sen-Dogan [1,*], Mehmet Alper Demir [1], Buket Sahin [1], Ender Yildirim [1,2,3], Gizem Karayalcin [1], Sebnem Sahin [1], Ege Mutlu [1], Taylan Berkin Toral [1], Ebru Ozgur [1], Ozge Zorlu [1] and Haluk Kulah [1,3,4]

[1] Mikro Biyosistemler A.S., 06530 Ankara, Turkey
[2] Department of Mechanical Engineering, Middle East Technical University, 06800 Ankara, Turkey
[3] METU MEMS Center, 06530 Ankara, Turkey
[4] Department of Electrical and Electronics Engineering, Middle East Technical University, 06800 Ankara, Turkey
* Correspondence: begum.sendogan@mikrobiyo.com.tr

Supplementary Material

1. Focusing of Fluorescent Beads inside the Microfluidic Channels

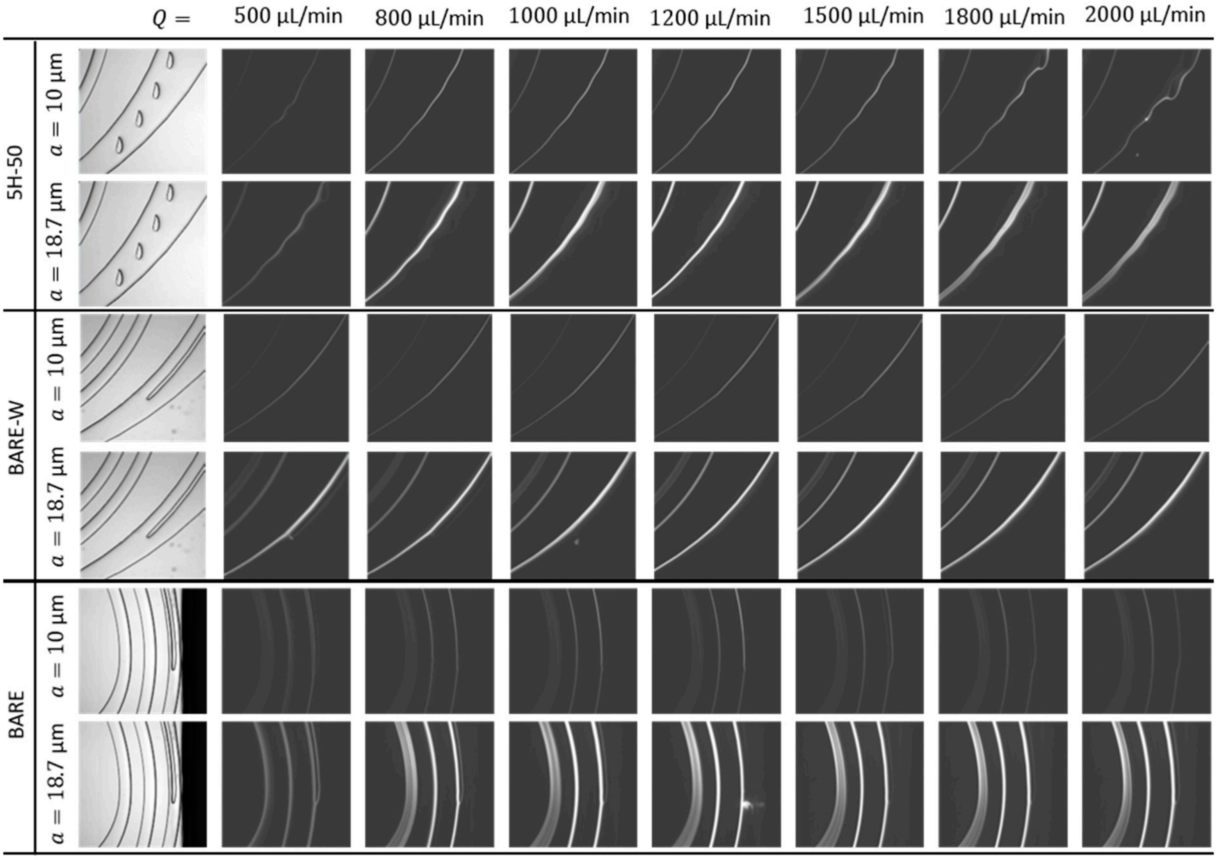

Figure S1. Bright field and fluorescent images showing the focusing lines for 10 µm beads and 18.7 µm beads along the different channel designs at different flow rates.

2. Immunofluorescent Characterization of Enriched CTCs

After the CTCs were enriched by the 5H-50 widening spiral channel with hydrofoils, CTCs collected at the product outlet were investigated by immunofluorescent (IF) staining.

Here, characterization of BT-474 cells processed through the channel the is presented. BT-474 enriched cell suspension collected at the product outlet of the spiral channel was centrifuged at 300 x g for 10 min, supernatant was removed and cells were fixed with 2% formaldehyde solution in PBS for 10 min at room temperature. After fixation, cells were washed 3 times at 1000 x g for 10 min. Then, cell pellet was transferred onto a poly-l-lysin coated slide in 30-40 µl PBS and smeared onto slide using the side of a pipette tip.

After all the liquid evaporated, permeabilization Buffer (200 µl, 0.1% Triton-X-100, Thermo Scientific, 8511, in PBS) was added onto each cell spot and incubated for 10 min at room temperature. Permeabilization buffer was removed by tapping slides onto a tissue and slides were washed 3 times for 5 min with wash Buffer (0.05% Tween-20, Sigma, P1379, in PBS, PBS-T). Then, blocking buffer (200 µl, 2% BSA, Sartorius, 03-010-1B, in PBS) was added and incubated for 1 hour at room temperature.

After blocking buffer was removed by tapping onto a tissue and antibody cocktail was prepared in 1% BSA in PBS and added onto cell spot without washing of blocking buffer. Antibody cocktail (Anti-Estrogen Receptor [EPR4097] (Anti-ER, Abcam, ab167610, 1:200), Anti-Progesterone Receptor [SP2] (Anti-PR, Abcam, ab239793, 1:200); they were conjugated with Alexa Fluor 488 (Conjugation Kit, Lightning Link, Abcam, ab236553), Alexa Fluor 555 Anti-HER2 [EP1045Y] (Abcam, ab281782, 1:200), Alexa Fluor 647 Anti-CD45 [EP322Y] (Abcam, ab200317, 1:200) and Alexa Fluor 750 Anti-pan Cytokeratin [AE-1/AE-3] (Anti-CK, Novus Biologicals, NBP2-33200AF750, 1:200)) was added onto each cell spot and incubated for 1 hour at room temperature. At the end of the antibody incubation, DAPI was added and incubated for 1 min and slides were washed for 5 min 3 times. Finally, slides were cover slipped with mounting medium (ProLong Dioamond Antifade mountant, Invitrogen, P36970) and waited until mounting medium was fully dry.

Slides were scanned with Bioview Allegro Plus (Rehovot, Israel) automated fluorescent microscope. Fig. S2 shows a region on the slide where one intact BT-474 cell and several WBCs are observed with different antibodies.

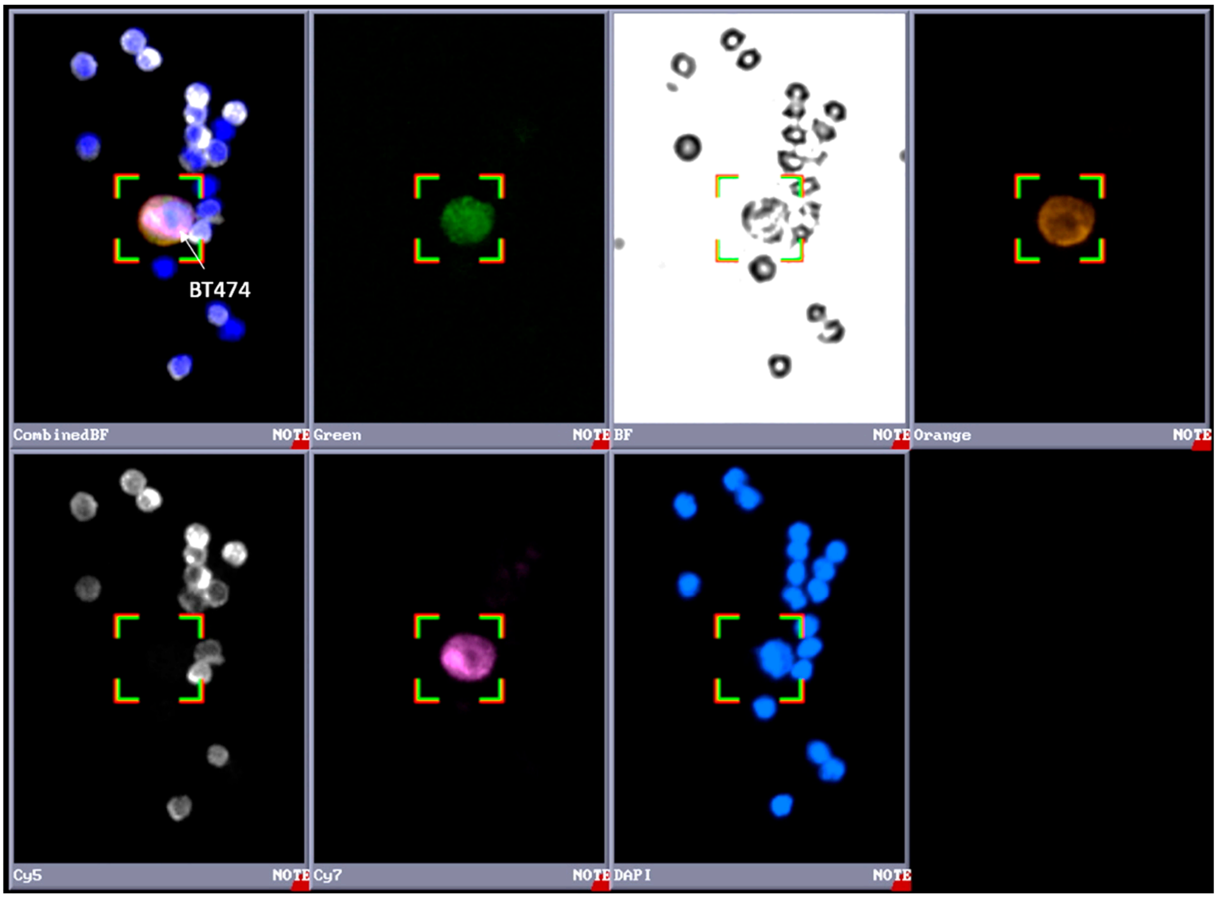

Figure S2. IF staining of BT-474 cells and white blood cells. BT-474 cells are ER/PR+, HER2+, CK+, DAPI+ and CD45-. Channels; Green: ER/PR, BF: Bright Field, Orange: HER2, Cy5: CD45, Cy7: pan Cytokeratin and DAPI.
